# Genome-wide transcriptional response of an avian pathogenic *Escherichia coli *(APEC) *pst *mutant

**DOI:** 10.1186/1471-2164-9-568

**Published:** 2008-11-28

**Authors:** Sébastien Crépin, Martin G Lamarche, Philippe Garneau, Julie Séguin, Julie Proulx, Charles M Dozois, Josée Harel

**Affiliations:** 1 Groupe de Recherche sur les Maladies Infectieuses du Porc (GREMIP), Université de Montréal, Faculté de médecine vétérinaire, C. P. 5000, Saint-Hyacinthe, Québec, J2S 7C6, Canada; 2 Institut National de la Recherche Scientifique, INRS-Institut Armand-Frappier, Laval, Québec, Canada

## Abstract

**Background:**

Avian pathogenic *E*. *coli *(APEC) are associated with extraintestinal diseases in poultry. The *pstSCAB*-*phoU *operon belongs to the Pho regulon and encodes the phosphate specific transport (Pst) system. A functional Pst system is required for full virulence in APEC and other bacteria and contributes to resistance of APEC to serum, to cationic antimicrobial peptides and acid shock. The global mechanisms contributing to the attenuation and decreased resistance of the APEC *pst *mutant to environmental stresses have not been investigated at the transcriptional level. To determine the global effect of a *pst *mutation on gene expression, we compared the transcriptomes of APEC strain χ7122 and its isogenic *pst *mutant (K3) grown in phosphate-rich medium.

**Results:**

Overall, 470 genes were differentially expressed by at least 1.5-fold. Interestingly, the *pst *mutant not only induced systems involved in phosphate acquisition and metabolism, despite phosphate availability, but also modulated stress response mechanisms. Indeed, transcriptional changes in genes associated with the general stress responses, including the oxidative stress response were among the major differences observed. Accordingly, the K3 strain was less resistant to reactive oxygen species (ROS) than the wild-type strain. In addition, the *pst *mutant demonstrated reduced expression of genes involved in lipopolysaccharide modifications and coding for cell surface components such as type 1 and F9 fimbriae. Phenotypic tests also established that the *pst *mutant was impaired in its capacity to produce type 1 fimbriae, as demonstrated by western blotting and agglutination of yeast cells, when compared to wild-type APEC strain χ7122.

**Conclusion:**

Overall, our data elucidated the effects of a *pst *mutation on the transcriptional response, and further support the role of the Pho regulon as part of a complex network contributing to phosphate homeostasis, adaptive stress responses, and *E. coli *virulence.

## Background

In *Escherichia coli *and many other bacterial species, the Pho regulon is activated when cells face phosphate limitation, whereas its expression is inhibited in excess of phosphate. The two-component system (TCS) PhoR/PhoB responds to environmental phosphate concentration variations and has been shown to control expression of at least 47 genes [1,2]. PhoR is an inner membrane sensor protein that responds to periplasmic orthophosphate (P_i_) concentrations and PhoB is the response regulator of the Pho regulon. PhoR is activated when the P_i _concentration is below 4 μM. Under P_i_-limited conditions, Phospho-PhoB binds to specific DNA sequences known as Pho-Boxes, located within Pho-dependent promoter regions to either induce or repress gene transcription. The *pstSCAB*-*phoU *operon encodes the Pst system and belongs to the Pho regulon. The Pst system encodes an ATP-binding cassette (ABC) transporter involved in the transport of P_i_. Importantly, mutations in the Pst system result in constitutive expression of the Pho regulon, regardless of environmental phosphate availability, and affect virulence of many pathogenic bacteria [1].

Extraintestinal pathogenic *Escherichia coli *(ExPEC) strains are associated with various diseases, including urinary tract infections (UTIs), newborn meningitis (NBM), abdominal sepsis and septicemia [3]. In poultry, APEC strains are a frequent cause of extraintestinal infections, collectively called colibacillosis. In addition, *E. coli *O78 strains can also cause diseases in hosts other than poultry, such as swine, sheep, and humans [4].

We have previously shown that inactivation of the *pst *operon in porcine and avian ExPEC strains resulted in constitutive expression of the Pho regulon and rendered the strains avirulent [4,5]. Moreover, it was reported that the *phoB *gene of the avian pathogenic *E*. *coli *(APEC) χ7122 strain is expressed during infection [6]. In accordance, in ExPEC strains, other PhoB regulated genes were shown to be expressed *in vivo *[7,8]. A number of reports have described an association between the Pst system, the Pho regulon and bacterial virulence [1]. Although inactivation of *pst *genes has been shown to affect the virulence of a number of bacterial pathogens, the mechanisms underlying this attenuation have not been elucidated.

Microarray studies have been conducted to understand how *E. coli *K-12 responds to growth in phosphate-rich or phosphate-limited conditions in *phoB *mutant strains [9,10]. Moreover, proteome profiles of cells grown under phosphate-rich or phosphate-limited conditions revealed that the overall phosphate response of *E. coli *may comprise up to 400 genes [11]. However, these studies investigated non-pathogenic *E*. *coli *K-12 grown in phosphate-limiting conditions. To understand global responses resulting from a mutation in the Pst system, which constitutively activates the Pho regulon, and its relationship with APEC virulence, the transcriptional profile of the APEC χ7122 strain was compared with its isogenic Pst mutant (K3) using the Affymetrix GeneChip^® ^*E. coli *Genome 2.0 Array. The Pho regulon is clearly not a simple regulatory circuit for controlling phosphate homeostasis; it is part of a complex network important for both bacterial virulence and the global stress response. Regulatory changes incurred due to inactivation of the Pst system resulted in modulation of genes involved in cell surface modifications, production of fimbrial adhesins, and protection against environmental stresses in the APEC mutant. These regulatory changes are likely to contribute to its reduced virulence and increased sensitivity to environmental stresses which may be encountered during host infection.

## Methods

### Bacterial strains, media and growth conditions

The APEC strain χ7122, the χ7122 Δ*pstCAB *mutant (K3), and the complemented χ7122 Δ*pstCAB *mutant (CK3) were previously described [4,12,13]. The χ7122 Δ*fim *strain χ7279 was generated by allelic exchange using suicide vector pDM915 as described by Marc *et al*. [12]. *E*. *coli *strains were grown at 37°C in Luria-Bertani (LB) medium. Antibiotics or supplements were used at the following final concentration, when required: 40 μg/ml for nalidixic acid (Nal), 50 μg/ml for kanamycin (Kan), 12.5 μg/ml for chloramphenicol (Cm) and 40 μg/ml for 5-bromo-4-chloro-3-indolylphosphate di-sodium (XP).

### Alkaline phosphatase assay

Alkaline phosphatase activity was measured as described previously with slight modifications [4]. Briefly, cells grown under different conditions were adjusted to an optical density of 1.0 at 600 nm (OD_600_), and 4 μg/ml of *p*-nitrophenyl phosphate (Sigma) was added to cells permeabilized by 50 μl of 0.1% SDS and 50 μl of chloroform. Color development was monitored at 420 nm and alkaline phosphatase activity was expressed in Miller units (MU), calculated as follows: 1 000 × [OD_420 _- (1.75 × OD_550_)]/*T *(min) × *V *(ml) × OD_600_].

### RNA isolation

RNA extractions were performed using four biological replicates of cultures of strains χ7122 and K3. Briefly, overnight cultures grown at 37°C in LB broth were diluted 100-fold into 5 ml of LB broth and were allowed to grow to mid-log phase (OD_600 _0.6). RNA samples were extracted with the RiboPure™-Bacteria Kit (Ambion, Austin, TX), according to the manufacturer's recommendations, with the exception that DNAse I treatment was performed twice. RNA concentration and purity were measured using the Nanodrop ND-1000 spectrophotometer (NanoDrop Technologies, Wilmington, DE) and the 2100 Bioanalyzer (Agilent Technologies, Santa Clara, CA). Quantitative RT-PCR (qRT-PCR) was performed on each RNA sample using a Smart Cycler (Cepheid, Sunnyvale, CA) to detect any DNA contamination. For this purpose, primers targeting the *rpoD *gene were used. RNAs were stored at -80°C for future use.

### cDNA synthesis and biotinylation

Ten μg of RNA dissolved in 20 μl of RNAse-, DNAse- and pyrogen-free water (Sigma) were supplemented with 2 μl of the GeneChip^® ^Eukaryotic Poly-A RNA Control Kit (Affymetrix, Santa Clara, CA) and converted into cDNA using SuperScript II and random hexanucleotide primers (Invitrogen, Carlsbad, CA), according to the manufacturer's instructions. Following cDNA synthesis, 2 μl of 1 mg/ml of RNAse A (Sigma) were added to the reaction mix and samples were incubated at 37°C for 30 min to degrade remaining RNA. cDNAs were purified using Microcon YM-30 centrifugal filters (Milipore, Billerica, MA). Concentration and purity were measured using the Nanodrop ND-1000 spectrophotometer. A range between 3 to 7 μg of cDNAs was fragmented using DNAse I (Ambion, Austin, TX), at concentration of 0.5 U/μg of cDNA, and by incubating at 37°C for 10 min. Fragmentation was stopped by heating the reaction mixture at 98°C for 10 min. Fragmented cDNAs were 3' biotinylated using GeneChip DNA Labelling Reagent (Affymetrix, Santa Clara, CA) at 7.5 mM and 60 U of Terminal Deoxynucleotidyl Transferase (Promega, Madison, WI) at 37°C for 60 min. The reaction was stopped by adding 2 μl of 0.5 M EDTA (Sigma). A gel-shift assay on 14% polyacrylamide gel using ImmunoPure NeutrAvidin (Pierce Chemical, Rockford, IL) was monitored to determine biotin incorporation.

### DNA microarray hybridization and analysis

The cDNAs were hybridized onto the Affymetrix GeneChip^® ^*E*.*coli *Genome 2.0 Array (Affymetrix, Santa Clara, CA) as recommended by the manufacturer . Hybridizations were performed at the Génome Québec Innovation Centre (McGill University, Montréal, Canada). Data were processed using the robust multiarray average algorithm (RMA) for normalization, background correction and expression value calculation [14]. Expression levels obtained from four independent biological replicates were compared using FlexArray 1.1 software [15]. Robustness of the data was further enhanced by the EB (Wright and Simon) algorithm and p-value calculation. Since the RMA algorithm diminished the false positive rate and compressed the fold change, a 1.5-fold change cut-off value was used for determination of the differentially expressed genes [16]. Functional classification was done according to the TIGR's Comprehensive Microbial Resource (CMR) [17]. Pathogen-associated ORFs were classified as such, since they represent sequences corresponding to one or more of the three pathogenic *E*. *coli *genomes (EDL933, Sakai and CFT073), but which are absent from the genome of *E*. *coli *K-12 strain MG1655.

### Quantitative RT-PCR

The QIAGEN QuantiTect^® ^SYBR^® ^Green RT-PCR kit was used for qRT-PCR according to the manufacturer's instructions. Primer pairs were designed using the PrimerQuest software from Integrated DNA Technologies  to yield PCR products varying between 80 to 200 bp. The *tus *gene was used as a housekeeping control. Each qRT-PCR run was done in triplicate and for each reaction, the calculated threshold cycle (*C*t) was normalized to the *C*t of the *tus *gene amplified from the corresponding sample. The fold-change was calculated using the 2^-ΔΔCt ^method [18]. Sequences of primers used for qRT-PCR analysis are available in additional file 1.

### *In silico *search for Pho box(es)

We used the DNA Pattern search program available on the Colibri^© ^website . The search was performed by using the pattern n [at]!c!c!g [at]!a!g!g!gn!g!g [tg] [ta] [ac]a [tc]nnn!g, where "n" represents any nucleotide, characters between square brackets " []" means an ambiguity and "!" before a character indicates the negation of this position. This pattern was designed based on different *E. coli *Pho box sequences presented by Blanco *et al*. [19]. We performed the DNA pattern search on the *E*. *coli *K-12 MG1655 whole genome, with no mismatch allowed, narrowing our search to the 200 bp upstream of predicted start codons. We also used the gene list containing predicted PhoB binding sites using the Pho box weight matrix elaborated by Yuan *et al*. [20]. Searches were performed against the *E. coli *MG1655, EDL933, Sakai, and CFT073 genomes which are represented on the Affymetrix GeneChip^® ^*E. coli *Genome 2.0 array.

### Sensitivity of *E. coli *strains to reactive oxygen intermediate (ROI)-generating agents

Sensitivity to oxidative stress generating agents was determined by an agar overlay diffusion method on LB plates (1.5% agar) as described by Sabri *et al*. [21]. Briefly, overnight cultures grown in LB broth were adjusted to an OD_600 _of 0.5. Then, 100 μl of each culture were suspended in molten top agar (0.5% agar) and poured over the agar plates. Filter paper disks (6 mm diameter; Beckton Dickinson) were added to the surface of the solidified overlays and 10 μl of hydrogen peroxide (30%), plumbagin (53 mM), phenazine methosulfate (PMS) (15 mM) or phenazine ethosulfate (PES) (15 mM) were spotted onto the disks. The plates were then incubated overnight at 37°C and following growth, the diameters of inhibition zones were measured.

### Yeast Cell Aggregation Assay

The yeast aggregation assay was derived from a micro-hemagglutination assay in 96-well round-bottom plates [13]. Briefly, cultures were grown to mid-log phase (OD_600 _0.6) in LB broth at 37°C with shaking (180 rpm) (conditions which were used for transcriptional analyses) or for 48 h without shaking at 37°C to enhance expression of type 1 fimbriae. Bacterial cells were centrifuged, and pellets were suspended in phosphate buffered saline (PBS, pH 7.4) to an initial suspension of approximately 2 × 10^11 ^cells/ml. Samples were then serially diluted two-fold in microtiter wells, and equal volumes of a 3% commercial yeast suspension were added to each of the wells. After 30 min of incubation on ice, yeast aggregation was monitored visually, and the agglutination titer was recorded as the most diluted bacterial sample giving a positive aggregation reaction. The Δ*fim *type 1 fimbriae mutant strain χ7279 was used as a negative control.

### Preparation of fimbrial extracts and Western blotting

Following growth of cultures with agitation at 37°C in LB to mid-exponential growth phase (OD_600 _0.6), fimbrial extracts were prepared and Western blotting was performed as described previously [22]. Briefly, fimbrial extracts were separated by sodium dodecyl sulfate (SDS)-15% polyacrylamide gel electrophoresis in minigels as previously described by Laemmli [23]. Proteins were transferred to nitrocellulose membranes (Bio-Rad) using a Mini Trans-Blot electrophoretic cell (Bio-Rad) for 60 min at 100 V. The membrane was blocked with StartingBlock (Pierce) supplemented with 0.05% Tween 20 (Pierce). Incubations with primary (1:5000) and secondary (1:25 000) antibodies were carried out for 1 h at room temperature. SuperSignal West Pico chemiluminescent substrate (Pierce) was used for detection. Primary antibodies, raised against type 1 fimbriae from *E. coli *strain B_AM_, were used, and react specifically with type 1 fimbriae from different APEC strains [24].

### Electron microscopy

Cells for electron microscopy were grown as described above for microarray experiments. A glow-discharged Formvar-coated copper grid was placed onto a drop of bacterial culture for 1 min to allow the cells to adsorb. The excess of liquid was then removed using a filter paper, just before a drop of 1% phosphotungstic acid (negative stain) was placed onto the grid. Samples were left to air dry and viewed using a Phillips EM300 transmission electron microscope.

### Microarray accession numbers

Microarray data are available at the National Center for Biotechnology Information Gene Expression Omnibus database  under accession number GSE9178.

## Results and discussion

### Microarray design to identify differentially expressed genes in the APEC *pst *mutant

To assess the effects of a *pst *mutation as well as Pho regulon activation on APEC strain χ7122, a transcriptional profiling approach was used. Strain χ7122 and its isogenic *pst *mutant (K3) were grown in a phosphate-rich (LB) medium. In this medium, strains χ7122 and K3 grew well, and they showed similar growth curves (data not shown). The alkaline phosphatase activity of PhoA is commonly used to evaluate the activation state of the Pho regulon [25]. The Pho regulon induction reaches its maximal rate at mid-log phase of growth in strain K3 as determined by PhoA activity (200 MU), whereas it was repressed in the wild-type parent strain χ7122 (3 MU). Thus, in the K3 strain, the Pho regulon is highly activated even during growth in phosphate-rich LB medium.

The Affymetrix GeneChip *E*. *coli *genome 2.0 Array contains oligonucleotides corresponding to four *E*. *coli *genomes (*E*. *coli *K-12 MG1655, *E*. *coli *enterohemorrhagic EDL933 and Sakai and extraintestinal *E*. *coli *(ExPEC) CFT073). Avian pathogenic *Escherichia coli *(APEC), a frequent cause of extraintestinal infections in poultry, are categorized as ExPEC. Although the genome sequence of the χ7122 APEC strain is not yet available, it is now known that the APEC O1:K1 strain genome shares 90% similarity with the CFT073 ORFs [26]. This suggests that probes on the GeneChip could represent at least in part the genome of χ7122. In addition, to determine the representative gene targets on the array that were present in strain χ7122, hybridization of χ7122 genomic DNA revealed that, among the 20,366 ORFs represented on the DNA array, 5751 were specific to the χ7122 genome. The non-K-12 ORFs were considered as pathogen-associated ORFs.

### Significant transcriptional changes in the K3 pst mutant

**Figure 1:**
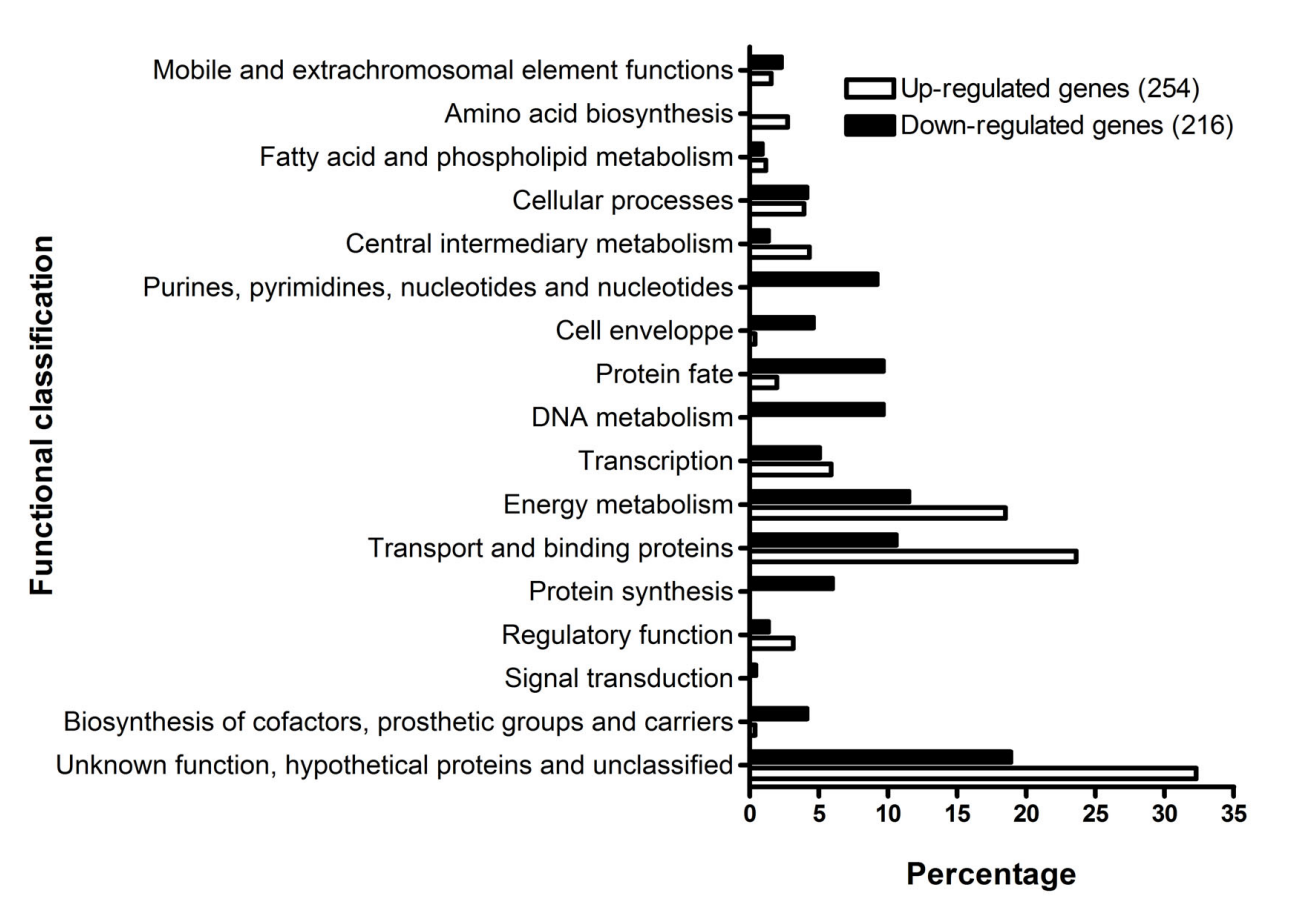
**Functional classification of the differentially expressed genes.** White and black bars represent the up- and down-regulated genes, respectively. The x-axis represents the percentage of the functional class according to the number of genes that were up- and down-regulated.

The transcriptomic study identified 470 genes that were differentially expressed by at least 1.5-fold in the *pst *mutant strain K3 when compared to the APEC strain χ7122, with a p-value of ≤ 0.05 and an estimated false discovery rate (FDR) of 2.71%. Globally, 254 genes were up- and 216 were down-regulated. Specific subsets of differentially expressed genes of known physiological relevance or putative function are discussed below and presented in Tables 1 and 2. A complete list of the differentially expressed genes is available online (see additional files 2 and 3). These tables contain genes that are discussed below and represent significant change at the transcriptional level occurring in Pho regulon activation. The known genes of the Pho regulon were induced varying from 145- to 2-fold. Besides those, the *gadW *gene showed the highest induction (6.8-fold), whereas the greatest repression was observed for the *yhcN *gene (-7.8-fold). Functional classification of the differentially expressed genes (Fig. 1) indicated predominant transcriptional changes among genes associated with cellular homeostasis and metabolism. Genes that were up-regulated included those encoding proteins of unknown function, transport and binding proteins, energy and central intermediary metabolism, and transcription (Table 1). Genes that were down-regulated also included the unknown function genes, as well as those involved with protein fate; protein synthesis; DNA metabolism; purine, pyrimidine, nucleoside and nucleotide pathways; and cell envelope proteins (Table 2). In addition, 5 small RNAs (sRNA) were differentially expressed. As expected, expression patterns of the genome-wide transcriptional response observed in the *pst *mutant in phosphate-rich medium shared similarities with transcriptomic analyses of non-pathogenic *E*. *coli *K-12 during phosphate starvation [9,10]. However, in contrast to *E. coli *K-12 which demonstrated marked phosphate limitation by early stationary phase (OD_600 _of 0.9), the APEC *pst *mutant exhibited an early maximal induction of the Pho regulon following growth to mid-log phase (OD_600 _0.6) in LB medium [10]. In addition, many genes associated with stress responses and metabolic functions were differentially expressed by the APEC *pst *mutant when compared to its wild-type parent.

**Table 1:** Up-regulated genes in K3 strain

**Functional class and gene name**^a,b,c,d,e,f,g^	**Operon**^c,d,e,f,h^	**Known or predicted function**	**Fold change**
**Transport and binding protein**			
*artI*	***art****P****IQM***^d^	Arginine-binding periplasmic protein 1	1.74
*artJ*^c^		Arginine-binding periplasmic protein 2	1.78
*feoB*	***feo****A*^c^***B***	Ferrous iron transport protein	2.19
*glnH*^c^	***glnHPQ***^d^	Glutamine ABC transporter periplasmic-binding protein	1.57
*mdtE*^e^	***mdtE****F*	Multidrug resistance efflux transporter	1.64
*oppA*^c^	***oppABC****DF*	Periplasmic oligopeptide-binding protein	2.66
*phnC*^b,c^	***phnCDEFGHIJKLMNOP***	Phosphonates transport ATP-binding protein	145.81
*phoE*^b,c^		Outer membrane phosphoporin protein	85.15
*potE*	*speF*-***potE***	Putrescine-ornithine antiporter	2.56
*pstS*^b,c^	***pstS****CA****B*-*phoU***	High-affinity phosphate-specific transport system	60.97
*srlA*^c^	***srlAEBD*-*gutM*-*srlR***^c^**-*gutQ***	PTS system, glucitol/sorbitol-specific IIC2 component	4.61
*ugpB*^b,c,d^	***ugpBAEC***^d^***Q***	sn-glycerol 3-phosphate transport system periplasmic binding protein	40.00
*yeaN*	***yeaN****O*	Putative amino acid/amine transport protein	2.91
*yhiD*^d,e^		Predicted Mg(2+) transport ATPase inner membrane protein	2.35
**Energy metabolism**			
*dhaK*	***dhaKL****M*	Dihydroxyacetone kinase	1.85
*grxB*		Glutaredoxin 2	1.85
*hcp*	***hcp*-*hcr***	Hydroxylamine reductase	1.63
*melA*	***melAB***	Alpha-galactosidase	3.24
*nagB*	***nagB****ACD*	Glucosamine-6-phosphate deaminase	1.83
*napH*	***nap****FDAG****HB****C*-*ccmABCDEFGH*	Quinol dehydrogenase membrane component	2.56
*nrfA*	***nrfABCD****EFG*	Cytochrome c552/nitrite reductase, formate-dependent	3.74
*talA*^c,d^		Transaldolase	1.57
*treA*^c,d^		Periplasmic trehalase	1.67
*ulaB*	***ula****A****B***^c^*C****DE****F*	L-ascorbate-specific enzyme IIB component of PTS	2.46
*ulaG*		Predicted L-ascorbate 6-phosphate lactonase	2.04
**Protein fate**			
*hdeA*^d,e^	***hdeAB***^d,e^	Acid-resistance protein	2.10
*iraP*^c,d^		Anti-adaptor protein for σ^S ^stabilization	4.42
**Cellular processes**			
*amn*^b,c^		AMP nucleosidase	6.08
*katE*^d^		Catalase HPII/hydroperoxidase HPII(III)	1.95
*sodC*^f^		Superoxide dismutase (Cu-Zn)	1.75
*ycgV*		Predicted adhesin	4.01
**Transcription**			
*chaB*	***chaB****C*	Cation transport regulator	1.75
*gadE*^e^		DNA-binding transcriptional activator	3.60
*gadX*^d,e^		DNA-binding transcriptional dual regulator	2.15
*gadW*^d,e^		DNA-binding transcriptional activator	6.84
*iciA*^b,c^		Chromosome replication initiation inhibitor protein	2.00
*phoB*^b,c^	***phoBR***	DNA-binding response regulator in two-component regulatory system with PhoR (or CreC)	36.23
*yhiF*^e^		Predicted transcriptional regulator	1.66
**Central intermediary metabolism**			
*gadA*^c,d,e^		Glutamate decarboxylase alpha	2.57
*gadB*^c,d,e^	***gadBC***	Glutamate decarboxylase beta	4.91
*phoA*^b,c^	***phoA*-*psiF***	Alkaline phosphatase	112.67
*sufA*^e^	***sufABCDSE***	Iron-sulfur cluster assembly scaffold protein	1.91
**Cell enveloppe**			
*yibD*^b^		Putative glycosyl transferase	45.95
**Fatty acid and phospholipid metabolism**			
*cdh*		CDP-diacylglycerol phosphotidylhydrolase	4.28
*hdhA*^d^		7-alpha-hydroxysteroid dehydrogenase	1.52
**Unknown function**			
*c0754*^g^		Hypothetical protein	2.12
*c0778*^g^	***c0778***-***speF***-***potE***	Hypothetical protein	2.43
*c0902*		Hypothetical protein	1.65
*c1013*		Hypothetical protein	1.83
*c1153*^c,g^		Hypothetical protein	2.35
*c1435*^g^		Hypothetical protein	2.26
*c2837*^g^		Hypothetical protein	1.53
*c4182*		Hypothetical protein	1.76
*phoH*^b,c^		Conserved protein with nucleoside triphosphate hydrolase domain	5.95
*psiE*^b,c^		Phosphate-starvation-inducible protein	1.84
*ytfK*^b^		Hypothetical protein	6.65
*z0950*^g^		Unknown	1.58
**Biosynthesis of cofactors, prosthetic groups and carriers**			
*iucD*^g^	*iucABC****D****-iutA*	Lysine/ornithine N-monooxygenase	1.76
**Regulatory function**			
*dps*^d,f^		DNA protection during starvation conditions	1.87
*isrA*		Small antisense RNA	1.64
*sgrS*		Small antisense RNA	1.98
*rybA*		Small antisense RNA	1.59
*rygC*		Small antisense RNA	1.50
*yddV*	***yddV*-*dos***	Predicted diguanylate cyclase	1.80

^a ^The first up-regulated gene of the operon is shown, ^b ^Members of the Pho regulon ^c ^Indicates the presence of Pho box in the gene promoter, ^d ^Genes belonging to RpoS regulon, ^e ^Genes involved in acid stress response, ^f ^Genes involved in oxidative stress response, ^g ^Pathogen-associated ORF, ^h ^Genes in bold are up-regulated in K3 strain.

**Table 2:** Down-regulated genes in K3 strain

**Functional class and gene name**^a,b,c,d,e,f^	**Operon**^b,c,d,e,g^	**Known or predicted function**	**Fold change**
**Transport and binding protein**			
*dctA*		C4-dicarboxylate transport protein	-2.03
*fadL*		Long-chain fatty acid transporter	-1.69
*proV*	***proVWX***	Glycine betaine transporter subunit	-2.30
**Energy metabolism**			
*fucI*^b^	*f****uc****P****IK****UR*	L-fucose isomerase	-1.59
*glpD*^c^		Aerobic glycerol-3-phosphate dehydrogenase	-3.56
*glpE*^b^	***glpEGR***	Thiosulfate sulfurtransferase	-2.04
*glpT*	***glpTQ***	sn-glycerol-3-phosphate transporter	-2.54
*glpX*	***glp****FK****X***	Fructose 1,6-bisphosphatase II	-1.51
*grxA*^e^		Glutaredoxin 1	-2.19
*trxB*^b,e^		Thioredoxin reductase, FAD/NAD(P)-binding	-1.88
*trxC*^b,e^		Thioredoxin 2	-1.87
**Protein fate**			
*c1935*^f^		Chaperone protein (F9 fimbriae)	-3.72
*dnaK*	***dnaKJ***	Molecular chaperone	-3.23
*grpE*		Heat shock protein	-1.68
*hscB*	***hscBA***-***fdx***-*iscX*	Co-chaperone with HscA	-1.81
*hslO*		Hsp33-like chaperonin	-1.59
*hslV*	***hslVU***	ATP-dependent protease peptidase subunit	-2.68
*htpG*^b^		Heat shock protein 90	-1.91
*ibpA*	***ibpAB***	Heat shock chaperone	-2.63
*slpA*^d^		FKBP-type peptidyl-prolyl cis-trans isomerase (rotamase)	-1.60
*ydhJ*	***ydhIJK***	Undecaprenyl pyrophosphate phosphatase	-2.05
**Protein synthesis**			
*dinD*		DNA-damage-inducible protein	-1.90
*lexA*	***lexA***-***dinF***	Regulator for SOS regulon	-2.14
*nrdA*	***nrdAB***	Ribonucleotide-diphosphate reductase alpha subunit	-1.58
*recN*		DNA repair protein/recombination and repair protein	-2.80
*rplR*	*rplNXE-rpsNH-**rpl**F**R**-**rpsE**-**rpmD**-**rplO**-secY-rpmJ*	50S ribosomal protein L18	-1.59
*rplW*	*rpsJ-**rpl**CD**WB**-**rpsS**-**rplV**-rpsC-**rplP**-**rpmC**-****rpsQ***	50S ribosomal protein L23	-1.91
*umuD*	***umuDC***	DNA polymerase V, subunit D	-1.72
**Purines, pyrimidines, nucleosides and nucleotides**			
*carA*^b^	***carAB***	Carbamoyl-phosphate synthase small subunit	-2.02
*guaB*	***guaBA***	Inositol-5-monophosphate dehydrogenase	-2.33
*purH*^b^	***purHD***	Bifunctional phosphoribosylaminoimidazolecarboxamide formyltransferase/IMP cyclohydrolase	-3.01
*purl*		Phosphoribosylformyl-glycineamide synthetase	-3.33
*purM*	*purMN*	Phosphoribosylaminoimidazole synthetase	-2.62
*pyrL*	***pyrLB****I*	Orotate phosphoribosyltransferase	-1.96
**Cell envelope**			
bcsZ	***bcs****AB****Z****C*	Endo-1,4-D-glucanase	-1.58
*eptA*		Predicted metal dependent hydrolase	-1.68
*rfaJ*	***rfa****QGPSBI****J****YZ*-*waaU*	Lipopolysaccharide 1,2-glucosyltransferase	-1.68
*rffC*	*rfe*-***rff****A****CD****E****GH****MT*-*wzxE*-*wzyE*-*wzzE*	Lipopolysaccharide biosynthesis protein/TDP-fucosamine acetyltransferase	-1.66
*yeiU*	***yei****R****U***	Undecaprenyl pyrophosphate phosphatase	-1.57
**Fatty acid and phospholipid metabolism**			
*fabB*^b^		3-oxoacyl-(acyl carrier protein) synthase	-1.51
*yieE*^b^	***yieE****F*	Predicted phosphopantetheinyl transférase	-1.53
**Transcription**			
*basR*	***basRS***^b^	DNA-binding response regulator in two-component regulatory system with BasS	-1.68
*crl*		Curlin genes transcriptional activatory protein	-2.52
*fimB*		Type 1 fimbriae regulatory protein/Inversion of on/off regulator of fimA	-1.66
*pdhR*^b^	***pdhR*-*aceEF*-*lpdA***	Transcriptional regulator for pyruvate dehydrogenase complex	-3.42
*rpoD*	*rpsU***-***dnaG***-*rpoD***	RNA polymerase sigma factor	-1.65
**Cellular processes**			
*c1936*^f^		Type 1 fimbrial protein (homologue)	-3.23
*fimA*^c^	***fimAIC****DFGH*	Major type 1 subunit fimbrin (pilin)	-2.10
*ydeQ*^b^		Predicted fimbrial-like adhesin protein (F9 fimbriae)	-1.57
*ydeR*	***ydeRST***^b^	Hypothetical fimbrial-like protein (F9 fimbriae)	-2.21
**Regulatory function**			
*oxyS*^e^		Global regulatory RNA	-1.82
**Central intermediary metabolism**			
*iscR*	***iscR****S****U****A*	NifU-like protein/Scaffold protein	-1.62
**Unknown function**			
*c3759*^b,f^	*parC-****c3759***	Hypothetical protein	-1.72
*pqiA*	***pqiA****B*	Paraquat-inducible membrane protein	-1.70
*yhcN*		Unknown	-7.82
*yjbC*	***ybjC***-*nfsA*-*rimK*-*ybjN*	Hypothetical protein	-1.54
*ybcU*		Bor protein homolog from lambdoid prophage DLP12	-1.62
*z5852*		Hypothetical protein	-1.52
**Biosynthesis of cofactors, prosthetic groups and carriers**			
*thiC*	***thiCEFSGH***	Thiamine biosynthesis protein	-3.30
**Mobile and extrachromosomal element functions**			
*hfq*	*yjeFE*-*amiB*-***mutL*-*miaA*-*hfq*-*hflX****K****C***	RNA-binding protein	-1.56
*z3347*^f^		Unknown protein encoded within prophage CP-933V	-1.65
*z1876*^f^		Putative endolysin of prophage CP-933X	-1.85

^a ^The first down-regulated gene of the operon is shown, ^b ^Indicates the presence of Pho box in the gene promoter, ^c ^Genes belonging to RpoS regulon, ^d ^Genes involved in acid stress response, ^e ^Genes involved in oxidative stress response, ^f ^Pathogen-associated ORF, ^g ^Genes in bold are down-regulated in K3 strain.

In contrast to the many differentially expressed *E. coli *genes involved in general metabolic or transport functions, only a few confirmed virulence- or stress-response associated genes demonstrated significant transcriptional changes in the *pst *mutant. Of these, genes involved in acid and oxidative stress response were modulated. Furthermore, genes encoding type 1 and F9 fimbriae were down-regulated in strain K3. However, the majority of the pathogen-associated ORFs identified are of unknown function. Among the 470 differentially expressed genes, 18 were not found in *E. coli *K-12 strain MG1655 and were considered as pathogen-associated ORFs. Of these, 14 were specific to CFT073 and 4 to the EHEC (EDL933 and Sakai) genomes. Overall we identified 18 pathogen-associated ORFs that are influenced by constitutive expression of the Pho regulon. However, the functions encoded by these ORFs are currently unknown.

Modulation of the pathogen-associated ORFs (Table 1 and 2) could contribute to the reduced virulence of strain K3 observed by Lamarche *et al*. [4]. Interestingly, the virulence-associated gene systems *sitABCD*, *iroBCDEN*, *iucABCD*-*iutA*, coding for metal transporters present in χ7122, that hybridized to the genomic array, were not differentially expressed in the attenuated *pst *mutant.

Blanco *et al*. showed that the C-terminal domain of PhoB interacts with a 22 bp region of dsDNA that consists of two direct repeats of 11 bp [19]. Each 11 bp repeat has a conserved 7 bp region (consensus, CTGTCAT) followed by a less conserved 4 bp segment. By *in silico *analysis and by using the list of genes identified in *E*.* coli *genomes by Yuan *et al*., a number of genes with predicted Pho boxes not associated with P_i _metabolism were identified among the differentially expressed genes [20]. Ninety-one genes or transcriptional units possessed putative Pho box(es), including the known Pho regulon members (Tables 1 and 2). Differentially regulated genes such as those involved in amino acid acquisition (*glnHPQ*, *artJ *and *oppABC*), energy metabolism (*srlAEBD*-*gutM*-*srlR*-*gutQ*, *treA*, *talA*, *ulaABCDEF*, *ulaG *and *glpD*), acid resistance (*gadA *and *gadBC*) and F9 fimbriae biosynthesis (*ydeTSR *and *ydeQ*) possess putative Pho box(es). Distribution of putative Pho regulon members across different functional classes supports the hypothesis that the Pho regulon overlaps and interacts with several other control circuits [1]. However, further studies will be required to establish whether these genes are directly regulated by PhoB.

### Validation of microarray results by qRT-PCR

**Figure 2:**
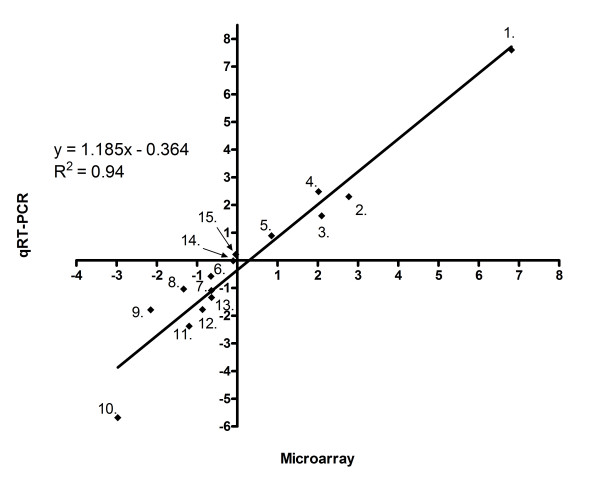
**Microarray results validation by qRT-PCR.** Five up-regulated genes, eight down-regulated genes and two non-differentially expressed genes are presented. Mean log_2 _ratios of the qRT-PCR experiments are plotted against the mean log_2 _ratios of the microarray experiments. Numbers on the graph refer to genes listed in Table 3.

Validation of microarray results was achieved using qRT-PCR. Fifteen genes representing a wide range of gene expression ratios (5 up-, 8 down-regulated and 2 non-differentially expressed genes) in K3 strain were selected for comparative qRT-PCR analysis (Table 3). Comparison of gene expression by microarray hybridizations and qRT-PCR demonstrated a very high level of concordance between the datasets, which is represented by a correlation coefficient of 0.94 and a Pearson correlation of 0.97 (Fig. 2).

**Table 3:** Genes used for microarray validation with qRT-PCR

No.	Gene	Gene title	Microarray Fold change (Log2)	qRT-PCR Fold change (Log2)
1	*phoA*	Alkaline phosphatase	6.816	7.600
2	*gadW*	DNA-binding transcriptional activator	2.773	2.303
3	*cdh*	CDP-diacylglycerol phosphotidylhydrolase	2.099	1.607
4	*ycgV*	Predicted adhesin	2.022	2.483
5	*yddV*	Diguanylate cyclase	0.851	0.890
6	*ydeQ*	Predicted fimbrial-like adhesin	-0.651	-0.573
7	*rpoD*	RNA polymerase sigma factor	-0.640	-1.087
8	*crl*	Curlin genes transcriptional activatory protein	-1.331	-1.037
9	*thiF*	Thiamine biosynthesis protein	-2.152	-1.787
10	*yhcN*	Hypothetical protein	-2.967	-5.680
11	*lexA*	Regulator for SOS regulon	-1.198	-2.377
12	*oxyS*	Oxidative stress regulator	-0.864	-1.777
13	*hfq*	RNA-binding protein	-0.638	-1.340
14	*yidB*	Hypothetical protein	-0.10	-0.013
15	*yeiR*	Predicted enzyme	-0.04	0.212

### Global stress response

Globally, the transcriptional profile of strain K3 indicated that in addition to up-regulating genes associated with scavenging pathways for phosphate acquisition and conservation, the *pst *mutant also demonstrated changes in expression of genes dealing directly with global stress. Many lines of evidence suggest that the Pho regulon and the stress response are interrelated [1]. The gene expression profile of strain K3 represents an exacerbated response in which the strain reacts to an inaccurate detection of phosphate-limiting conditions when, in fact, phosphate levels are actually abundant. This adaptive response entails the induction of different mechanisms to optimize the acquisition and the bioavailability of phosphate and to maintain the essential biochemical reactions active. In addition, during growth, the K3 mutant activates a generalized stress response adaptation.

### Genes associated with the RpoS regulon

The Pho regulon and the RpoS regulon are interrelated regulatory networks of the bacterial adaptive response [1]. RpoS is a sigma factor implicated in the cellular response to many stresses. It is also implicated in the stationary phase and the induction of genes in nutrient-limiting environments [27]. The *rpoS *gene is expressed under a variety of growth conditions, but regulation and RpoS production is largely dependent on post-transcriptional stability [27,28]. During exponential growth, in the K3 mutant, the *rpoS *gene was not differentially expressed. However, the RpoS-regulatory gene *iraP *was induced 4.2-fold. IraP encodes an anti-adaptor protein that enhances RpoS stability and accumulation by inhibiting its targeting to the ClpXP degradosome [29,30]. Moreover, the small regulatory RNA (sRNA) OxyS, that inhibits the translation of *rpoS*, was down-regulated (-1.82-fold). Bougdour and Gottesman have recently shown that the transcription of the *iraP *gene is promoted by ppGpp accumulation during phosphate starvation [30,31]. These results suggest that in strain K3, during exponential phase, the sigma factor RpoS stability is increased, leading to regulatory expression of numerous RpoS-dependent genes. Accordingly, 49 genes known to be regulated by the RpoS sigma factor are differentially expressed in strain K3 (Tables 1 and 2). Hence, in the exponential growth phase, strain K3 may alter its gene regulation to respond to phosphate limitation, which includes establishment of a general stress response. Quantification of the RpoS protein would further demonstrate the establishment of the RpoS response, at mid-log phase of growth, in the *pst *K3 mutant.

### Oxidative stress response

During aerobic growth, the cytotoxic by-products of the molecular oxygen metabolism, collectively known as reactive oxygen species (ROS), must be eliminated to reduce oxidative stress. It is generally thought that the primary endogenous source of ROS is the respiratory chain, namely the NADH dehydrogenase II, which can leak electrons to oxygen, thereby producing superoxide anion radicals (O_2_^-^•). Superoxide is normally detoxified through the activities of superoxide dismutases and catalases that dismute O_2_^-^• into molecular oxygen, hydrogen peroxide (H_2_O_2_) and eventually to H_2_O. However, if they are not rapidly detoxified, superoxide radicals can damage iron-sulfur clusters in enzymes, thereby releasing Fe^2+^, which can react with H_2_O_2 _and produce reactive hydroxyl radicals (HO•). HO• can eventually initiate the oxidation of proteins, lipids and DNA [32]. Several genes that belong to the RpoS regulon are involved in defense against oxidative stress (*katE*, *dps*, *sodC *and *xthA*) and/or OxyR (which controls *oxyS*, *katG*, *ahpCF*, *gorA*, *grxABC*, *dps *and *fur*) and SoxR (which notably controls *sodA*, *nfo *and *fur*) (for a review, see reference [32]). In strain K3, some genes involved in the oxidative stress response were found to be differentially expressed. Among those, the *dps *(DNA protection protein and ferritin-like protein), *sodC *(superoxide dismutase Cu-Zn), *grxB *(Glutaredoxin 2), *katE *(catalase) and *sufABCDSE *(Fe-S cluster assembly proteins) genes were up-regulated from 1.95- to 1.75-fold. The down-regulated genes (from -2.19- to -1.54-fold) were represented by *grxA *(glutaredoxin 1), *trxB *(thioredoxin reductase), *trxC *(thioredoxin 2), *pqiA *(paraquat-inducible protein) and *ybjC *(predicted inner membrane protein). In addition, the sRNA OxyS regulator was down-regulated (-1.82) in strain K3. OxyS has been proposed to play a role in protecting cells against the damaging effects of elevated hydrogen peroxide concentrations by controlling translation of > 40 genes associated with the oxidative stress response [30,33]. Other, regulators involved in oxidative stress, such as OxyR and SoxRS, were not differentially expressed in strain K3.

Since expression of some genes whose products exhibit antioxidant activities is modulated, the viability to oxidative stress of K3 cells was tested using different reactive oxygen intermediate (ROI)-generating agents. We used H_2_O_2 _and various superoxide generators, such as plumbagin, phenazine methosulfate (PMS) and phenazine ethosulfate (PES) to evaluate the sensitivity of the K3 *pst *mutant and its wild-type parent strain χ7122. Strain K3 was more sensitive to oxidative stress than the parent strain χ7122, and wild-type resistance levels to all ROI compounds, except for H_2_O_2_, were restored by complementation (strain CK3) (Table 4). These results show that the *pst *mutation affects bacterial resistance to oxidative stress, and modulation of certain genes implicated in the oxidative stress response is not sufficient to confer resistance to this stress. Based on the transcriptional data, strain K3 may already be subjected to increased oxidative stress during growth, and is likely less able to cope with additional stresses incurred from exogenous ROI-generating compounds. Furthermore, Moreau *et al*. have shown that glucose metabolism in non-growing cells starved for Pi generates oxidative stress [34,35]. Since oxidative stress is increased during infection of the host, a decreased capacity to resist ROS could explain, at least in part, virulence attenuation observed for *pst *mutants [4,5,7,36].

**Table 4:** Growth inhibition zone (mm) of APEC χ7122, K3 (pst mutant) and CK3 (complemented) strains to oxidative stress generating compounds^*a*^

**Strain**	**LB agar with:**			
	**Plumbagin**^*b*^	**H_2_O_2_**	**PES**	**PMS**
**χ7122**	8.5 ± 0.1	19.2 ± 0.2	15 ± 0.4	15.7 ± 0.4
**K3**	**10.7 ± 0.6**^*c*^	**23.0 ± 0.9**	**19.2 ± 0.5**	**20.7 ± 0.2**
**CK3**	8.7 ± 0.3	**21.9 ± 1.0**	15.1 ± 0.3	16.7 ± 0.2

^*a *^Data presented are the means ± the standard deviations of six independent experiments.

^*b *^Compounds used were 10 μl of plumbagin (53 mM), H_2_O_2 _(30% vol/vol), phenazine ethosulfate (15 mM) (PES), or phenazine methosulfate (PMS) (15 mM).

^*c *^Values indicated in bold text are significantly different (P < 0.05) compared to the mean of the wild-type strain.

### Acid stress response

The acid fitness island (AFI) contains genes encoding proteins that are known to provide protection against acid stress in *E. coli*. This broad acid response system helps the cell avoid self-imposed acid stresses that occur as a result of fermentation and enables the cell to survive to low pH conditions [37,38]. The microarray data demonstrate that the expression of the AFI genes increased significantly in strain K3. This includes the glutamate decarboxylase gene *gadA *(2.57-fold), the multiple transcriptional regulator genes that control expression of the glutamate dependent acid resistance (GDAR) system, *gadE *(3.60-fold), *gadX *(2.15-fold) and *gadW *(6.84-fold), the two chaperones *hdeAB *(2.10-fold for the *hdeA *gene), the multidrug resistance efflux transporter *mdtE *(1.64-fold) and the transcriptional regulator *yhiF *(1.66-fold). In accordance, the *gadBC *operon (4.91-fold for the *gadB *gene), which encodes the glutamate-decarboxylase and an antiporter, respectively, were also up-regulated. Regulation of the AFI and GDAR systems is extremely complex and multifactorial. The expression of these systems is controlled by a large number of regulators, including, among others, the EvgAS two-component system, the specific transcriptional regulators (GadX, GadW, YdeO, and GadE) and the RpoS sigma factor [39]. Up-regulation of genes involved in acid resistance suggests that the *pst *mutant may be subjected to increased acid stress during exponential growth. The effect of the Pho regulon on induction of the acid resistance response is likely to be indirect, since no putative Pho box(es) were found upstream of these regulators.

Previous reports have described the relationship between the Pho regulon and genes involved in acid stress resistance [9,10,40,41]. Transcriptional activation of the *gadA*/*BC*/E genes was observed in *E*. *coli *W3110 grown in low phosphate media [9,10]. In spite of the induction of acid stress genes, strain K3 was previously shown to be more sensitive to acid stress [4]. It is possible that the *pst *mutant is already subjected to a decreased internal pH from an accumulation of organic acids produced by the glycolytic pathway, which could induce acid response genes during exponential growth [40]. As such, strain K3 which may already incur endogenous acid stress, may therefore be less able to cope when subjected to decreased external pH and acid stress, as observed by Lamarche *et al*. [4]. Based on these data, it would be interesting to compare the pH of the culture medium and the intracellular pH of the wild-type and *pst *mutant during growth, to assess whether induction of these acid response genes may be specifically due to increased acidity during growth.

### Transport and binding proteins

In addition to Pho regulon members involved in the transport of phosphate and phosphorylated compounds, genes encoding amino acid and oligopeptide transport systems were up-regulated in strain K3. Indeed, the glutamine and the arginine transporters, encoded respectively by the *glnHPQ *operon and by the *artIQM*/*artJ *genes were up-regulated in strain K3 (1.57- and 1.74-fold, respectively). Down-regulation of the *argR *(-1.57-fold) gene, which encodes the transcriptional repressor of the arginine transporter, occurred at the same time as with the up-regulation of the arginine transporter *artIQM*/*artJ*. The oligopeptide transporter, encoded by the *oppABCDF *genes, was also induced (2.66-fold). This transporter is specific for small peptides of up to five amino acids. Besides their roles in proteins synthesis, amino acids can serve as nitrogen, carbon and ATP sources and they can protect cells from different stresses, such as acid stress [42]. Furthermore, genes encoding the glutamine, arginine and oligopeptide transporters possess putative Pho box(es), which suggest that the uptake of oligopeptides and amino acids via these transporters may be directly regulated by the Pho regulon.

### Energy metabolism

Some metabolic genes, involved in the catabolism of different carbon sources, were up-regulated. The *srlAEBD*-*gutM*-*srlR*-*gutQ *operon, involved in glucitol transport and metabolism and belonging to the phosphoenolpyruvate (PEP): carbohydrate phosphotransferase system (PTS), was induced in strain K3 (4.61-fold) [43]. In addition, up-regulation of genes enzymatically linked to the production of fructose-6-phosphate (*talA *and *nagB*) (1.57- and 1.8-fold, respectively) and glucose (*melA *and *treA*) (3.71- and 1.67-fold, respectively), and metabolism of dihydroxyacetone (*dhaKL*) (1.85-fold) and L-ascorbate (*ulaBDE *and *ulaG*) (2.46-fold) were also induced. These catabolic pathways link to the glycolytic pathway, and intermediates could take part in the tricarboxylic acid cycle. Interestingly, the small regulatory RNA (sRNA) SgrS, which inhibits synthesis of the PtsG glucose-specific PTS system and alleviates the effects of glucose-phosphate stress, was also up-regulated (1.98-fold), suggesting that the strain K3 has preferentially shifted to transport and metabolism of carbon sources other than glucose [44,45].

On the other hand, repression of genes involved in catabolism of glycerol-3-phosphate (G3P) as a carbon source, such as the G3P transporter encoded by the *glpTQ *genes (-2.54-fold) and the glycerol-3-dehydrogenase *glpD *(-3.56-fold) was observed. The Glp system mediates utilization of G3P or its precursors, glycerol and glycerophosphodiesters. Since uptake of G3P by the GlpTQ transporter leads to P_i _counter flow and an overall loss of P_i _from the cell, it is not surprising that this system is repressed in strain K3 [46]. By contrast, genes encoding the other G3P-specific transporter Ugp were highly up-regulated (40.00-fold). Accordingly, the *ugp *genes are members of the Pho regulon. Under phosphate starvation conditions, G3P is transported by the Ugp transporter, which leads to the use of G3P as a phosphate source [2]. Identification of putative Pho box(es) upstream the *srl *and *ula *operons, the *talA*, *treA*, and *ulaG *genes denotes a possible direct connection between these catabolic pathways and the Pho regulon. Furthermore, the presence of putative Pho boxes upstream of the *glpD *gene suggests that the Pho regulon inhibits the use of G3P as carbon source. Thus, as the *pst *mutant falsely senses phosphate limitation, it may alter carbon uptake and utilization pathways in an effort to conserve phosphate sources. In order to specifically determine differences in carbon source utilization and transport by the *pst *mutant compared to its wild-type parent, strains could be compared for their capacity to metabolize and grow on different carbon sources.

### Protein fate and synthesis

Many genes from the protein fate and synthesis functional classes were down-regulated in strain K3. These genes included those encoding components of the 30S and 50S ribosomal subunits and many chaperone genes involved in protein fate, such as those of the DnaK system and iron-sulfur cluster assembly. The *dnaKJ *(-3.23-fold), *grpE *(-1.68-fold) and *ibpAB *(-2.63-fold) genes comprise the DnaK system. This system is involved in many cytoplasmic events, such as folding of nascent polypeptide chains, rescue of misfolded proteins and assembly and disassembly of protein complexes [47,48]. Iron-sulfur cluster complexes play several important roles in cellular processes, such as iron storage, electron transfer and regulation of enzyme activity and gene expression [49]. However, genes involved in iron-sulfur cluster assembly were down-regulated in strain K3. They were represented by *hscAB*-*fdx *(-1.81-fold) and *iscU *(-1.62-fold) genes. Repression of the DnaK system and iron-sulfur cluster assembly genes suggests a decrease in cellular processes occurring in strain K3. Down-regulation of chaperones and ribosomal components suggest a reduction in cellular processes and a potential increase of misfolded peptides, which could be deleterious for cells and may contribute, at least in part, to virulence attenuation of the APEC *pst *mutant strain. Such changes are likely to be indirectly influenced by the Pho regulon, since there were no putative Pho box(es) identified upstream of these genes.

### Cell membrane components

In many bacteria, the Pho regulon was shown to be involved in regulation of some cell surface component modifications such as teichuronic acid, phosphate-free lipids, phospholipids and exopolysaccharides [1]. Among the differentially expressed genes in strain K3, 109 encode membrane components. Of these, 54 genes were up- and 55 were down-regulated (Tables 1 and 2, and additional files 2 and 3). Many of the up-regulated genes belong to the transport and binding protein functional class and include members of the Pho regulon that were increased by more than 15-fold. In contrast, the *proVWX *operon (-2.3 fold), involved in glycine betaine osmoprotector transport, demonstrated a decreased expression. This system is implicated in protection from osmotic shock [50]. Beside their role in energy metabolism, the *talA *(1.57-fold) and *treA *(1.67-fold) genes were also induced by osmotic stress under aerobic conditions [51,52]. This suggests that the *pst *K3 strain was faced with osmotic stress, and down-regulation of the *proVWX *operon could reduce protection from this stress.

Among the down-regulated genes, 7 are involved in lipopolysaccharide (LPS) biosynthesis including lipid A modification. Indeed, the *rffCDGH *(-1.66-fold) and the *rfaJ *(-1.68-fold) genes, respectively involved in the enterobacterial common antigen (ECA) biosynthesis pathway and LPS-core biosynthesis, were down-regulated. However, we previously did not observe any changes in O-antigen and/or ECA profiles by SDS-PAGE and Western blot analyses [4,53]. The results of this study suggest that subtle changes could indeed occur within the LPS structure in strain K3.

The *eptA *and *yeiU *genes, involved in lipid A modification, were also down-regulated (-1.68 and -1.57-fold, respectively). The *eptA *gene is the *Salmonella pmrC *ortholog and is involved in phosphoethanolamine (pEtN) covalent modification of lipid A [54]. The BasRS two-component system that regulates *eptA *was also down-regulated (-1.68-fold) in this study [54]. The *basRS *genes are orthologs of the *pmrAB *genes of *Salmonella enterica*, and PmrAB has been shown to be required for resistance to polymyxin B and other antimicrobial compounds [55]. In addition, we have determined that, in strain K3, the down-regulation of *yeiU *(*lpxT*), encoding an undecaprenyl pyrophosphate phosphatase involved in the biosynthetic origin of the lipid A 1-pyrophosphate, correlated with a decrease in the hexa-acylated 1-pyrophosphate form of lipid A compared to the APEC strain χ7122 [53,56]. Valvano MA and Raetz *et al*. have proposed that modification of lipid A might contribute to the fine-tuning of the lipopolysaccharide molecule, which is often the target of modifications that might provide adaptive advantages to changing environmental conditions [57,58]. LPS modification could contribute to the phenotypes observed in the *pst *mutant, such as decreased resistance to serum, acid and cationic antimicrobial peptides [4,5,53]. Furthermore, down-regulation of LPS modification genes may be a strategy used by the bacteria to optimize the P_i _availability. However, such cell surface modifications could play a role in the reduced virulence observed in *pst *mutants [1].

### Reduced expression of fimbrial genes and production of type 1 fimbriae

Fimbriae are adhesive organelles of paramount importance for successful bacterial recognition and colonization of specific host tissues. Genes involved in type 1 and F9 fimbriae biosynthesis were down-regulated in the K3 strain (Table 2). Indeed, genes involved in the biosynthesis of type 1 (major type 1 subunit *fimA*, the chaperone *fimC *and the *fimI *gene, whose function is not elucidated, and the regulator of type 1 fimbriae *fimB*) (-2.1 fold) as well as F9 fimbriae (*ydeTSR*, *ydeQ*, *c1936 *and *c1935*) (-3.72- to -1.54-fold) were repressed in strain K3. Type 1 and F9 fimbriae are important virulence factors involved in colonization and biofilm formation [59-63]. In APEC strains, type 1 fimbriae are preferentially expressed in air sacs, which are the primary infection site [64-66]. F9 fimbriae are homologous to type 1 fimbriae but are immunologically and functionally distinct [62]. However, the production of F9 fimbriae and the possible role of these fimbriae in APEC infections have not yet been investigated.

**Figure 3:**
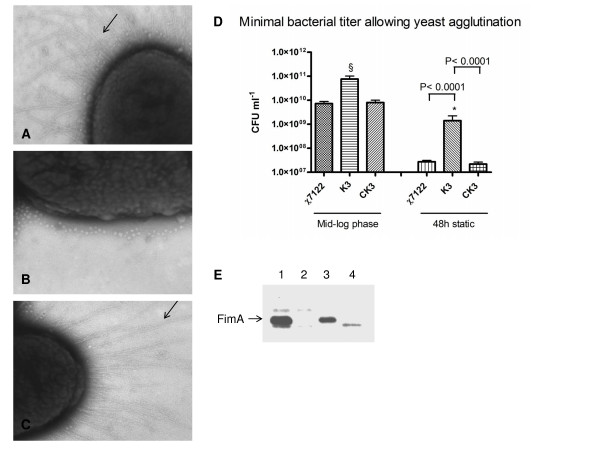
**Production of type 1 fimbriae by the strain K3.** A, B and C) Electron microscopy performed onto χ7122, K3 and CK3 strains, respectively, at 12 000×. Images show a typical field of view of bacteria and demonstrate that the K3 strain lacks fimbriae, compared to the χ7122 and CK3 strains. Arrow shows fimbriae on cell surface. D) Minimal bacterial titer allowing yeast agglutination. The χ7122 Δ*fim *(χ7279) strain was used as negative control and did not show agglutination (data not shown). The § symbol shows that at mid-log phase of growth, no agglutination was observed at the highest cell titer for the K3 strain, which was also observed for Δ*fim *strain χ7279 (data not shown). Asterisks indicate significant differences observed between bacterial concentration of the wild-type χ7122 and the *pst *mutant strain K3 (*P value *= 0.0001) as calculated by Student's t test. No difference was observed between the χ7122 and the CK3 strains. E) Western blot of protein extracts from strains χ7122 (lane 1), K3 (lane 2), CK3 (lane 3), and χ7279 (lane 4) using anti-FimA serum.

In order to investigate whether the decreased expression of fimbrial genes correlated with decreased fimbrial production, strain χ7122, the *pst *mutant K3 and the complemented mutant CK3 were grown under conditions used for microarray analyses and were examined by electron microscopy. Indeed, very few or no fimbriae were observed at the surface of cells of the K3 mutant, compared to wild-type strain χ7122 and the complemented strain CK3 (Fig. 3A,B,C). The type 1 fimbrial adhesin recognizes D-mannose receptors on cells, and mediates aggregation of yeast cells which are rich in mannose surface molecules [67].

When cultures of strains χ7122, the *pst *mutant K3, and the complemented CK3 were grown under conditions corresponding to those used in the microarray analyses, i.e. OD_600 _0.6, the K3 strain did not demonstrate any yeast aggregation even at the most concentrated bacterial cell suspension (approx. 1 × 10^11 ^cfu/ml), whereas χ7122 and the complemented mutant CK3 agglutinated yeast at titers at least 10-fold less (approx. 1 × 10^10 ^cfu/ml) (Fig 3D). When strains were cultured for 48 h without agitation, to enhance production of type 1 fimbriae, the K3 mutant agglutinated yeast cells, but 51-fold less than wild-type strain χ7122 and the complemented mutant (Fig. 3D). Hence, the *pst *mutant was shown to be considerably less able to produce type 1 fimbriae compared to its wild-type parent even under different growth conditions. To determine whether decreased yeast agglutination was specifically due to a reduction in production of the type 1 fimbrial adhesin, cell surface extracts were tested for production of the type 1 major fimbrial subunit, FimA, by Western blotting, and this confirmed an important reduction of FimA production in K3 strain, compared to χ7122 and CK3 strains (Fig. 3E).

Several studies have provided evidence that the expression of type 1 fimbriae is altered in response to environmental stress conditions such as high osmolarity, pH and temperature, and that expression is induced upon entry into stationary phase [68,69]. Putative Pho box(es) were identified upstream of the *ydeT *and *ydeQ *genes, which are involved in F9 fimbriae biosynthesis, whereas no putative Pho box(es) were observed upstream of the genes involved in type 1 fimbriae biosynthesis. Taken together, results indicate that in the *pst *mutant, the production of type 1 fimbriae is repressed, and this loss of adhesin expression could be indirectly controlled by PhoBR. It was previously shown that in APEC strains, type 1 fimbriae are preferentially expressed in the air sacs and lungs, where the air sacs are the primary site of initial infection [65,70]. Thus, in the K3 strain, in addition to other previously established phenotypes, down-regulation of adhesins such as type 1 fimbriae could affect the initial step of colonization and possibly contribute to decreased virulence.

## Conclusion

In conclusion, by assessing the global transcriptional response of a *pst *mutant, we determined that the effects of this mutation resulted in up-regulation of members of the Pho regulon, which are involved in phosphate uptake and metabolism. Transcriptional analyses also demonstrated the induction of a general stress response in the *pst *mutant, including genes involved in adaptation to acid stress, oxidative stress, and the general stress response (notably RpoS-regulated genes). Other changes included down-regulation of genes associated with cell surface composition. Phenotypic tests confirmed a reduced capacity of the *pst *mutant to produce type 1 fimbriae and a decreased capacity to resist oxidative stress. Altogether, our data provide further support that the Pho regulon is an important part of a complex network that encompasses not only phosphate homeostasis, but also adaptive responses to stress and altered regulation of a diversity of genes including virulence factors such as those encoding fimbrial adhesins.

## Abbreviations

Pst system: Phosphate specific transport system; EDTA: ethylene diamine tetraacetic acid; cDNA: complementary DNA; ORF: Open Reading Frame; bp: base pair; Fe^2+^: Ferrous iron; Fe-S: Iron-sulfur cluster; SDS-PAGE: Sodium Dodecyl Sulfate Polyacrylamide Gel Electrophoresis.

## Authors' contributions

SC designed and performed the transcript profiling experiments, carried out downstream data analysis and drafted the manuscript. MGL constructed the K3 and CK3 strain, participated in the study design and revised the manuscript. PG executed the bioinformatics analysis and revised the manuscript. JS carried out the characterization of fimbriae experiments. JP performed the oxidative stress experiments. CMD supervised the characterization of fimbriae and oxidative stress experiments, performed data analysis and revised the manuscript. JH participated in the conception and supervised the design of the study, performed data analysis and revised the manuscript. All authors read and approved the final manuscript.

## Supplementary Material

Additional file 1 **List of primers used in the qRT-PCR experiments.** Complete lists of primers used in the qRT-PCR experiments, including primer sequences are shown. Click here for file

Additional file 2 **Up-regulated genes in the K3 strain.** The complete list of genes up-regulated by at least 1.5-fold with a p-value of ≤ 0.05 and an estimated false discovery rate (FDR) of 2.71%, in the *pst *K3 strain. Probe number, gene name, fold change in Log2, and p-value were provided. Click here for file

Additional file 3 **Down-regulated genes in the K3 strain.** The complete list of genes down-regulated by at least 1.5-fold, with a p-value of ≤ 0.05 and an estimated false discovery rate (FDR) of 2.71%, in the *pst *K3 strain. Probe number, gene name, fold change in Log2, and p-value were provided. Click here for file
