# Supplementary material for: Genome-wide transcriptional response of an avian pathogenic Escherichia coli (APEC) pst mutant
Source: BMC Genomics. 2008 Nov 28;9:568. doi: 10.1186/1471-2164-9-568 (PMC2648988; doi:10.1186/1471-2164-9-568)
Supplement: Additional file 1 — List of primers used in the qRT-PCR experiments. Complete lists of primers used in the qRT-PCR experiments, including primer sequences are shown. [file 1471-2164-9-568-S1.doc]

| **Additional table 1. Primers used for the qRT-PCR experiments** | | |
| --- | --- | --- |
| **Genes** | **Primers** | **Sequence 5’→ 3’** |
| *phoA* | *phoA* F | CGCCAAATCCGCAACGTAATGACA |
|  | *phoA* R | TCGCCAATTTGCCCACAAGGATTC |
| *gadW* | *gadW* F | AGGATGTCGATGGCCAGACGATTA |
|  | *gadW* R | GTTGCGCAAACTGATGTGGCGTTA |
| *cdh* | *cdh* F | CTGATGTGCGCAAACAGCTGGATA |
|  | *cdh* R | AAATGGGCTGCGTTGTACCAGTTC |
| *ycgV* | *ycgV* F | TAGGCACAGGCAGCACCATTAAGA |
|  | *ycgV* R | ACTTCCGCTTCCAAGATCGACACT |
| *yddV* | *yddV* F | TGAAGAAGTATCGCGCCACGAAGT |
|  | *yddV* R | GCATGGGCAATTTCGCGTTTGAAG |
| *ydeQ* | *ydeQ* F | AATGTTGTTATGCCAACAGGCGGC |
|  | *ydeQ* R | TCAGTGGTGGCACCCGAAAGATAA |
| *rpoD* | *rpoD* F | ACGCCGATCGGTGATGATGAAGAT |
|  | *rpoD* R | TCGATACCGAAACGCATACGCAGA |
| *crl* | *crl* F | TCGATTGTCTGGCTGTATGCGTCA |
|  | *crl* R | ACACTCTTCCAGTCGCCTGCTTTA |
| *thiF* | *thiF* F | AGATAACCAGGAGCCAGAACGCAA |
|  | *thiF* R | ACTGGCTCGATTTACCGTCGAACA |
| *yhcN* | *yhcN* F | ACTCCATTGATGCTGCACAAGCAC |
|  | *yhcN* R | AGCTTCAGTAATCTGGTAGGCCGT |
| *lexA* | *lexA* F | TGTTGCAGGAAGAGGAAGAAGGGT |
|  | *lexA* R | GCAGGAAATCAGCATTCGGCTTGA |
| *oxyS* | *oxyS* F | AACCCTTGAAGTCACTGCCCGTTT |
|  | *oxyS* R | CGGATCCTGGAGATCCGCAAA |
| *hfq* | *hfq* F | GCTGCAAGGGCAAATAGAGTCTTT |
|  | *hfq* R | TTACTGTGATGAGAAACCGGGCGA |
| *yidB* | *yidB* F | ACAGGGCAATCAATCGGTTAGTGG |
|  | *yidB* R | AAGCTGTACTGGTATCCACGCCAA |
| *yeiR* | *yeiR* F | TGGATTTGCCGCGTCGCAATTTAG |
|  | *yeiR* R | TGATATCCTTGCCCGCTGTTCAGA |
| *tus* | *tus* F | CGATAACCTTTCGCAAGCAGCGTT |
|  | *tus* R | GGCAAATGACGATGCACCCATTCA |
